# Multispectral pattern recognition measures change in drusen area in age-related macular degeneration with high congruency to expert graders

**DOI:** 10.1038/s41598-022-11070-6

**Published:** 2022-05-06

**Authors:** Judy Nam, Angelica Ly, Michael Kalloniatis, Lisa Nivison-Smith

**Affiliations:** 1 Centre for Eye Health, University of New South Wales, Sydney, NSW 2052 Australia; 2 School of Optometry and Vision Science, University of New South Wales, Sydney, NSW 2052 Australia

**Keywords:** Macular degeneration, Prognostic markers, Medical imaging

## Abstract

Drusen are a hallmark lesion of age-related macular degeneration (AMD) and changes in their area and/or volume are strongly associated with disease progression. Assessment of longitudinal change in drusen size in clinical practice however is limited to a single commercial tool or manual inspection by clinicians. In this study we analysed change in drusen area in 33 eyes with intermediate AMD across two separate visits using a novel technique known as multispectral pattern recognition for *en face* retinal images from various imaging modalities (infrared (815 nm), fundus autofluorescence (488 nm) and green (532 nm) scanning laser ophthalmoscopy). We found 91% (30/33 eyes) agreement in the direction of drusen change for multispectral pattern recognition relative to expert graders who graded eyes as having drusen progression, regression or being stable. Multispectral pattern recognition showed 100% sensitivity (22/22 eyes) and 73% specificity (8/11 eyes). In comparison, we found only 70% (23/33 eyes) agreement in the direction of drusen change with a commercially available change analysis software, the Cirrus Advanced RPE Analysis relative to expert graders, with a sensitivity 64% (14/22 eyes) and specificity of 82% (9/11 eyes). Total drusen area or amount of change between visits had no significant effect on agreement. This suggests multispectral pattern recognition can quantify longitudinal change in drusen area from multimodal imaging with greater congruency to expert graders than a commercially available platform based on a single imaging modality. Considering the association of drusen area and disease progression, this method could aid clinical assessment and monitoring of AMD.

## Introduction

Age-related macular degeneration (AMD) is the leading cause of irreversible blindness in developed countries worldwide with severe vision loss occurring in late stages of disease. Examining change in drusen size^1^ and area^2,3^ over time is fundamental to managing patients with early AMD as both drusen progression and drusen regression are considered precursors to late AMD^4–6^. Specifically, correlations between large drusen area or volume have been associated with development of both non-neovascular and neovascular AMD^4,7–9^ and large drusen diameter is used as a point scale for calculation of progression risk to late AMD in the AREDS simplified scale^10,11^. Alternatively, loss of drusen and subsidence of surrounding retinal layer substructures, a process known as nascent geographic atrophy has been identified as a strong predictor for progression to geographic atrophy^12,13^.

A number of studies have explored methods to quantify drusen change in AMD utilising optical coherence tomography (OCT)^7,14–18^ or OCT angiography^19^. A commercially available tool is also currently available within the Cirrus Spectral domain (SD)-OCT (Carl Zeiss Meditec. Inc., CA, USA) known as the ‘Advanced RPE Analysis’ which indicates changes in drusen elevations in the RPE over time from OCT images^20^. These methods have been able to quantify longitudinal increases and decreases of change in drusen volume. However, several limitations have been associated with OCT quantification of drusen including segmentation errors and low sensitivity to small drusen volumes^21,22^.

Recently, a multimodal approach has been recommended to comprehensively visualise AMD and help determine the best clinical management based on structural disease markers^12^. For drusen, several advanced eye imaging modalities such as infrared imaging (IR)^23,24^, green scanning laser ophthalmoscopy^25^, fundus autofluorescence (FAF)^26,27^ and OCT^27–29^ have been shown to be useful in differentiating different drusen subtypes^27,30^ and a multimodal approach is suggested for improved accuracy of AMD staging^31,32^. Comparison of drusen segmentation between OCT and *en face* retinal images also show different advantages to each modality with the former useful for detection of large drusen areas while color fundus photographs (CFPs) are superior in detecting subtle changes in drusen^21,22^. A method which uses a multimodal approach to assess drusen change however has yet to be explored.

We recently developed a computational approach using multispectral pattern recognition which integrates *en face* images from multiple modalities into a single, user-friendly output^33^. This image analysis has been shown to accurately identify drusen with high specificity and potentially assess drusen change over time^33^.

However, it is not known how this analysis performs over the breadth of drusen changes which can occur over time (i.e. progression and regression) and how this approach compares to other methods of drusen change quantification currently used in clinical practice. Thus, the aim of this study was to determine the ability of multispectral pattern recognition to accurately quantify change in drusen area over time relative to expert graders and compare this to a commercially available, semi-automated drusen quantification method, the ‘Advanced RPE Analysis’ of the Cirrus SD-OCT.

## Methods

### Study population

Thirty-three eyes of 23 participants with bilateral intermediate AMD^1^ who attended the Centre for Eye Health, Sydney Australia for at least two visits were retrospectively analysed. The Centre for Eye Health is an intermediate-tier optometry clinic that provides advanced eye imaging and diagnostic services for referred patients with non-urgent ocular pathologies^34–36^. Written informed consent was obtained from all participants in accordance with the Declaration of Helsinki and approved by the Biomedical Human Research Ethics Advisory Panel of the University of New South Wales.

Participants were included if they had an initial macular assessment between January to December 2015 and a follow up visit any time before December 2020, were older than 55 years, and had no concomitant macular disease other than intermediate AMD. AMD classification was based on the Beckmann Initiative and the structural parameters of drusen and pigmentary abnormalities^1^. Eyes were excluded if no drusen was present or there was the presence of other AMD structural features reported in the patient record which could be confounded with drusen appearance including reticular pseudodrusen, hyperreflective foci and nascent geographic atrophy^6,37^. Participants also required the following images accessible in their patient file from each appointment: a central 45 degree color fundus photograph (Kowa WX 3D Nonmydriatic retinal camera; Kowa, Nagoya, Japan), a fundus autofluorescence image (FAF, Optos Panoramic 200Tx; Optos, Dunfermline, UK), a green scanning laser ophthalmoscopy image (Green532nm, Optos Panoramic 200Tx), a central infrared scanning laser ophthalmoscopy image (IR815nm, Spectralis Heidelberg Retina Angiograph 2; Heidelberg Engineering, Heidelberg, Germany) and a macular SD-OCT cube scan (Cirrus 6000; Zeiss, Carl Zeiss Meditec. Inc., CA, USA). With a peak reflectance of approximately 560 nm^25^, drusen appears bright on all imaging modalities. Whereas pigmentary abnormalities appear dark especially in green light due to absorption, which further ensured the distinction between the two structural parameters. Images were deemed suitable quality for inclusion if image brightness and contrast levels and/or the signal strength were of clinically acceptable quality and images were free of artefacts. For participants where image quality was suitable at the first visit but unsatisfactory at the immediate subsequent visit, images from the following appointment were considered if available. Hence mean follow up time in the study reflects time between images assessed for the study and not necessarily time between consecutive AMD assessments.

### Expert grading of drusen change

The ground truth for direction of change in drusen area was defined according to the patient reports from the Centre for Eye Health. Clinical decisions in these reports are made by the examining optometrist upon reviewing images from multiple modalities. Reports are finalised and signed off by a reviewing principal optometrist and/or consultant ophthalmologist. All clinicians involved in report writing have expertise in interpretation of retinal imaging. For the purposes of this study, all eyes were reviewed by two expert graders as part of the routine clinical assessment at the Centre to reflect displaying drusen progression, no change (stable) or drusen regression.

### Multispectral pattern recognition analysis

**Figure 1:**
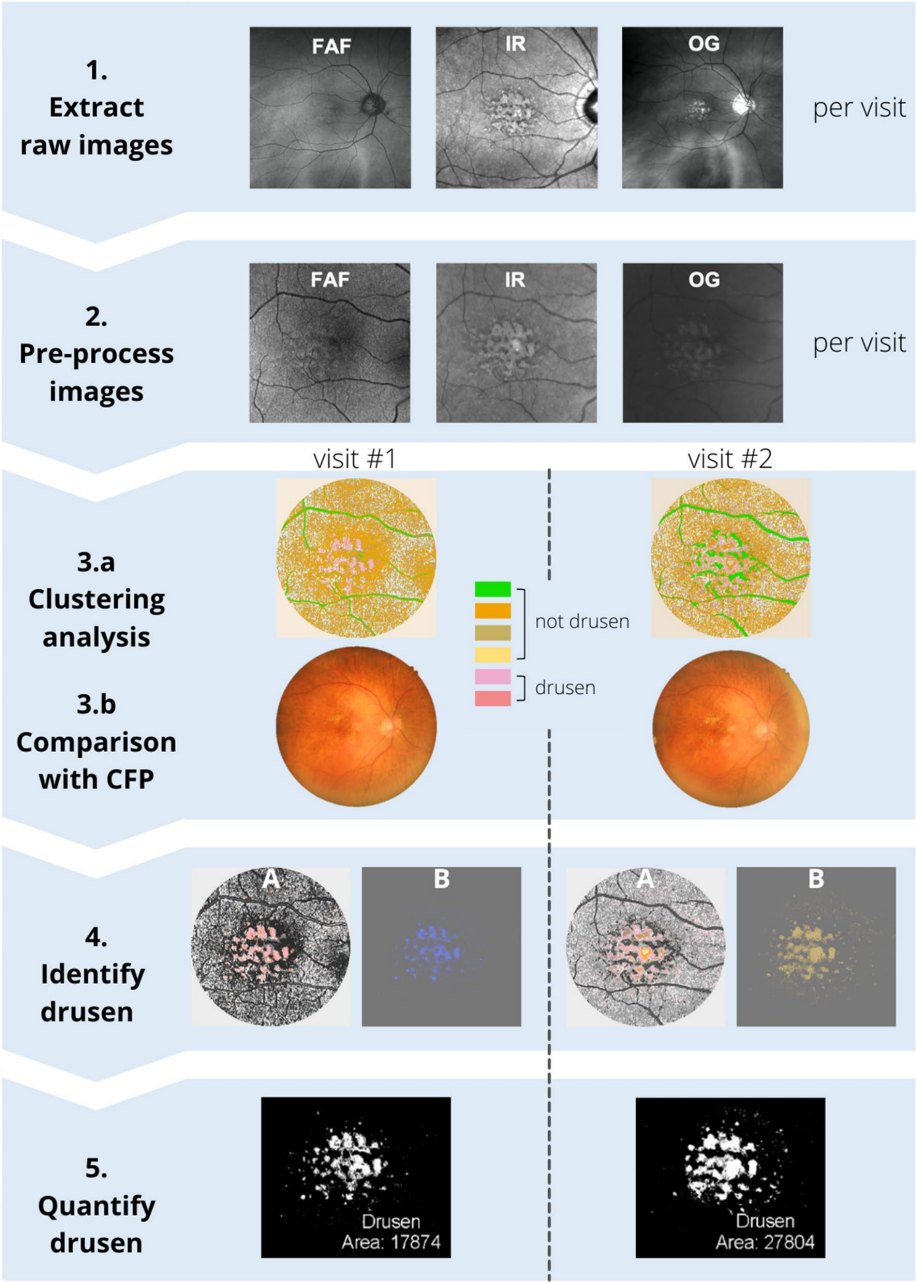
Summary of the methodology. Step 1: Extraction of raw images from each imaging modality at each visit. Note that images vary in field of view due to use of different imaging modalities. Step 2: Pre-processing of images as described in Ly et al.^33^ Images are registered, background corrected, and contrast enhanced. Step 3.a: Clustering analysis using pattern recognition is performed across the three images yielding a single multispectral image where each color represents a distinct spectral theme class. Step 3.b Spectral theme classes assigned as drusen/not drusen using reference color fundus photograph annotated for drusen by clinical experts. Step 4: Identification of spectral theme classes attributed to drusen from the pseudocolored image. (A): Spectral theme classes identified as drusen were thresholded from all other theme classes in the pseudocolored image and (B) Drusen spectral theme classes were merged to create a duochrome image. Step 5: Quantification of drusen. Total number of pixels attributed to drusen were quantified from duochrome images at each visit and drusen change calculated as a percentage difference between the two visits. CFP: Color fund photograph, FAF: fundus autofluorescence, IR: infrared, OG: green scanning laser ophthalmoscopy images.

**Figure 2:**
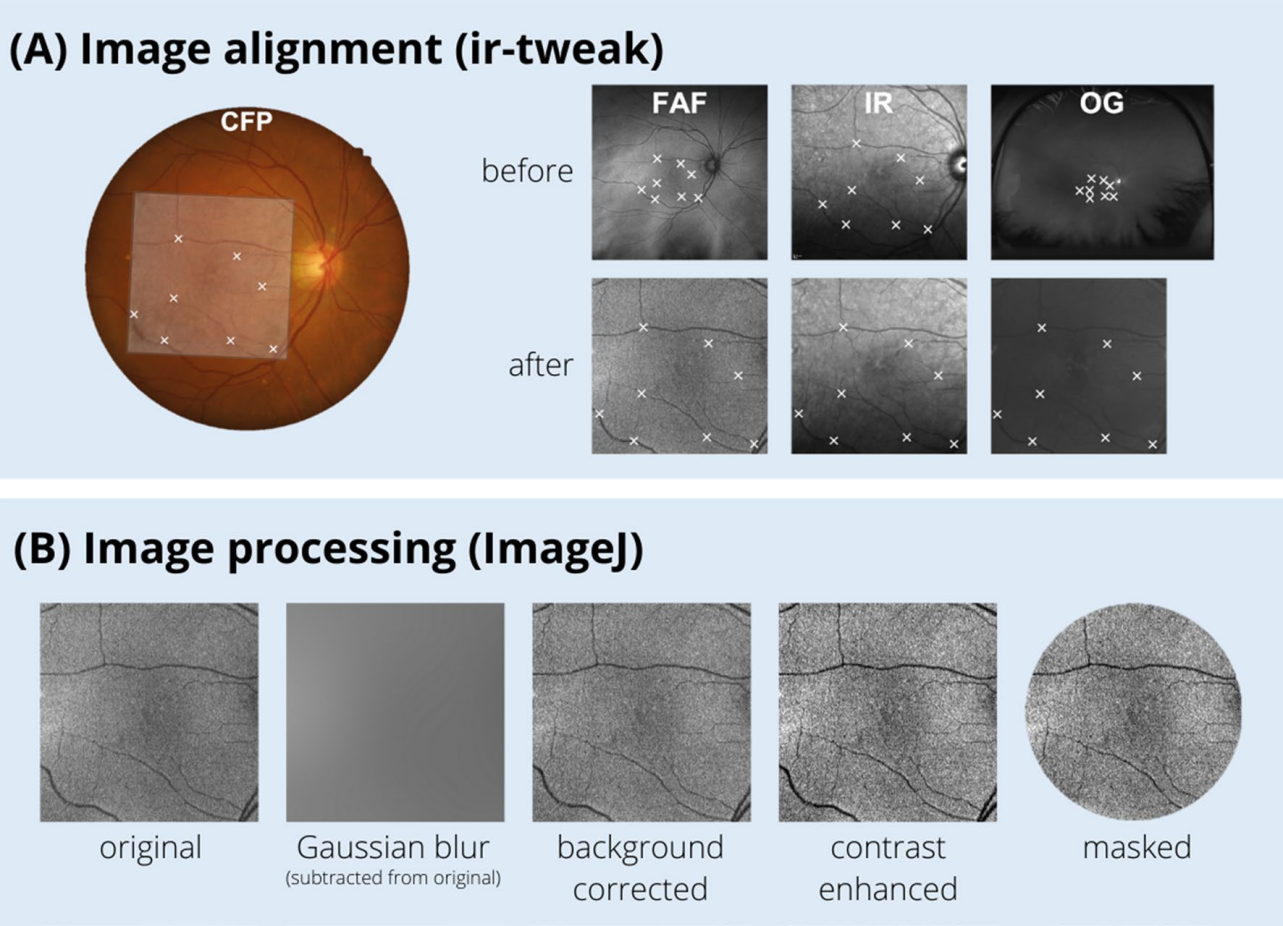
(**A**) Image alignment was performed on FAF, IR815nm and Green532nm scanning laser ophthalmoscopy using ir-tweak such that the eight blood vessel bifurcation reference points were matched to the CFP. (**B**): Images were processed using ImageJ to ensure useful classification of retinal architecture: background corrected by subtracting the Gaussian blurred image from the original, contrast enhanced and masked to remove areas beyond the macula.

Multispectral pattern recognition analysis (Fig. 1) was performed as described previously by Ly et al^33^. Fundus autofluorescence, IR815nm and Green532nm scanning laser ophthalmoscopy images for each eye at each visit were extracted from patient data files (Fig. 1, Step 1). Extracted images were manually aligned in ir-tweak (University of Utah, Salt Lake City, UT, USA). Image alignment was required to ensure that when images were overlapped in subsequent multispectral analysis, all features would be aligned across the images. Alignment of FAF, IR815nm and Green532nm scanning laser ophthalmoscopy images was done to the reference CFP using eight blood vessel bifurcation reference points (Fig. 2A). Image alignment was then confirmed in final images by manual inspection and flicker comparison, a technique of viewing images in quick succession to detect differences.

Images were converted to grey scale (expressed as pixel values), then pre-processed in *ImageJ* (National Institutes of Health, Bethesda, MD, USA), and masked to include the central macula area only (Fig. 1, Step 2)^33^. Specifically, images were background corrected to ensure useful classification of retinal architecture by subtracting the Gaussian blurred image from the original in ImageJ. Images were then contrast enhanced using the enhance contrast function with a threshold set at 0.4% saturation and masked using a template to remove areas beyond the macula (Fig. 2B).

Using an unsupervised k-means clustering algorithm and separability statistics, the images from FAF, IR815nm and Green532nm scanning laser ophthalmoscopy were classified using multispectral pattern recognition with *PCI Geomatica* (PCI Remote Sensing, Ontario, Canada). The final classification resulted in spectral theme classes sharing similar pixel values derived from the three spectral input channels (FAF, IR815nm and Green532nm, all converted to a greyscale and processed as noted above) separated with an accuracy of at least 83%.

The k-means classification process began with 16 maximum classes (k = 16), i.e., 16 classes would be found using a 0.01 minimum threshold. After each iteration, the number of clusters were reduced to achieve the defined minimum transformed divergence (D_T_) value of ≥ 1.5. At each iteration, pairwise comparisons were undertaken for each of the classes the k-means algorithm derived. If the separability of any of the clusters did not meet our D_T_ criterion, the k-means algorithm was repeated with the two clusters with the lowest D_T_ separability merged. This process was repeated until all cluster separability was at D_T_ ≥ 1.5. A criterion of D_T_ ≥ 1.5 was set based on previous optimisation^33^ which is analogous to a greater than 83% probability of correct classification (Fig. 1, Step 3A). Details of classification process are further discussed elsewhere^38–42^.

All theme classes from the pseudocolored image were then assigned as being drusen or not drusen in accordance with a reference CFP which was manually annotated for drusen as well as hyper- and hypopigmentary abnormalities by two clinical experts as described in Ly et al.^33^ (Fig. 1, Step 3B).

All spectral theme classes identified as drusen were isolated from pseudocolored images by thresholding by color using *Adobe Photoshop* (Adobe Inc., Mountain View, CA, USA). All other spectral theme classes were converted to greyscale to form the background (Fig. 1, Step 4A). The average blur function was then applied to create a duochrome image resulting in the combination of all theme classes attributed to drusen into one color and the background to another color (Fig. 1, Step 4B).

Duochrome images were binarized with *ImageJ* to create an 8-bit image whereby the background (containing no drusen) was assigned black, and the foreground (containing drusen) was assigned white (Fig. 1, Step 5). Drusen was then quantified by determining the pixel count of the foreground. The overall difference in drusen area between visits was calculated and quantified as a percentage. Classification as drusen progression, regression or stable was based on > 5% change, >  − 5% change and − 5% to 5% change in drusen area respectively.

### Quantification of drusen from cirrus advanced RPE analysis

The Advanced RPE Analysis of the Cirrus SD-OCT (Cirrus 6000; Zeiss, Carl Zeiss Meditec. Inc., CA, USA) is a commercially available drusen change analysis tool that quantifies drusen area and volume by measuring the elevations in the RPE from a macula cube scan. Details of the algorithm are described extensively elsewhere^15^. For each eye at each visit date, macula cube scans were inspected for image quality including a minimum signal strength > 7 and the absence of image artefacts, particularly in the outer retina. The segmentation of the RPE layer was also assessed and manually corrected where necessary. Drusen change was then extracted from the Advanced RPE Analysis screen as the percentage difference quoted by the software for the ‘Area in 5 mm Circle’ centred on the fovea.

### Comparative analysis

Sensitivity and specificity of multispectral pattern recognition and the Cirrus Advanced RPE Analysis was determined by comparing to expert grading. If an eye was categorised as stable by expert graders using flicker comparison but demonstrated a percentage change by an analysis greater or less than 5%, then it was considered a false positive for drusen progression and drusen regression, respectively.

## Results

### Study population

A total of 33 eyes were included in the study. Eleven eyes were graded as having drusen progression, 11 eyes having relatively no change in drusen area and 11 eyes having drusen regression. The demographics and ocular characteristics between each sub-group were not significantly different (Table 1). An average follow up time of 1.3 years was noted for all eyes and was consistent with current clinical recommendations for intermediate AMD to be reviewed at least annually^43,44^.

**Table 1:** Demographics of each drusen change sub-group.

	Drusen change sub-group			
	Regression	Stable	Progression	*P* Value
Eyes, *n*	11	11	11	
**Age (years)**				
Mean	75	70	70	0.056^a^
Range	65–86	64–80	60–77	
Sex (Female:Male)	73:27	73:27	45:55	0.308^b^
**BCVA (logMAR)**				
Mean	0.07	0.11	0.11	0.631^a^
Range	0.00–0.34	− 0.08–0.32	0.00–0.32	
**Refractive error (D)**				
Mean	− 0.08	1.29	0.38	0.293^a^
Range	− 3.58–2.42	− 1.67–4.17	− 4.17–4.50	
**Follow up time (years)**				
Mean	1.5	1.1	1.3	0.340^a^
Range	0.5–3.5	0.5–1.5	0.5–2.2	

Abbreviations: D, diopters.

^a^Analysed using one-way ANOVA. ^b^Analysed using chi-square test.

### Spectral theme classes identified in multispectral pattern recognition

**Figure 3:**
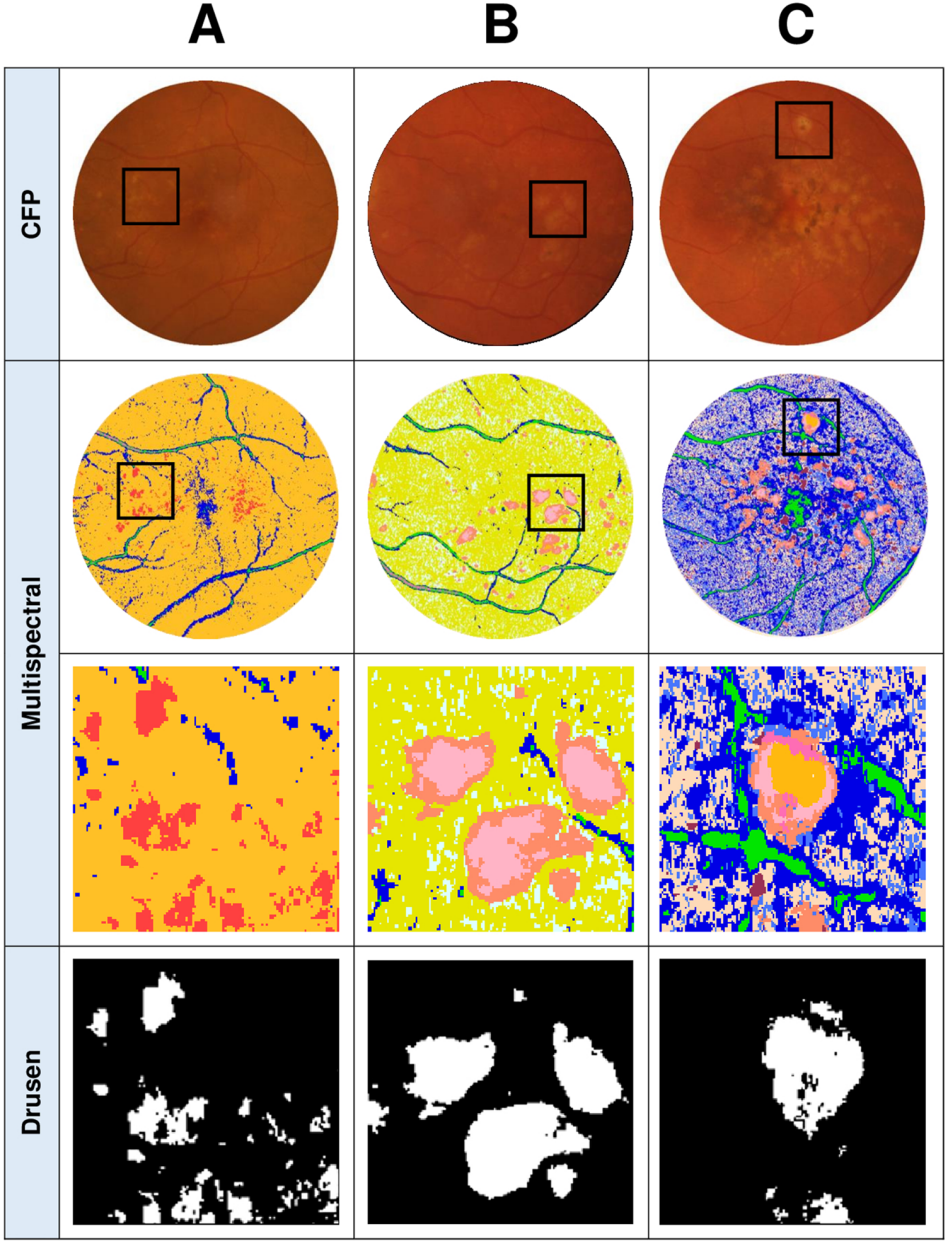
CFPs and OCT line scans from three different eyes and their corresponding pseudocolored images (derived from multispectral image analysis) showcasing the different types of drusen spectral theme classes that emerged. (**A**): An example of an eye where a single drusen spectral theme classes (red) emerged from the pseudocolored image. (**B**): An example where two separable spectral theme classes for drusen (pink and peach) emerged which were then pooled for the quantification of drusen area. (**C**): An example of an eye which yielded multiple spectral theme classes in the pseudocolored image for drusen (orange, light pink, and peach) as well as other AMD lesions such as pigmentary abnormalities (dark pink, maroon) that can be seen on the CFP and OCT (colored arrows indicate each spectral theme class relative to OCT line scan). Only the spectral theme classes identified as drusen were pooled for the quantification of drusen area (final row). Boxes in CFP and multispectral images indicate locations of magnified images. Lines through magnified CFP images indicate location of OCT line scan.

On average, there was 2 ± 1 different spectral theme classes associated with drusen identified per multispectral pattern recognition analysis which was consistent between visits for each eye. In eyes where multiple spectral theme classes could be assigned to drusen, these spectral theme classes were generally adjacent to one another and clearly identifiable as drusen from corresponding CFPs (Fig. 3B). In eyes where different AMD lesions were present within drusen areas such as pigmentary abnormalities, multispectral analysis successfully assigned these lesions to a separate spectral theme class which ensured they were not pooled in the final analysis (Fig. 3C)*.* Examples of pooling of spectral theme classes identified as drusen for subsequent quantification can be seen in Fig. 3, final row.

### Sensitivity and specificity of multispectral pattern recognition to drusen change

**Figure 4:**
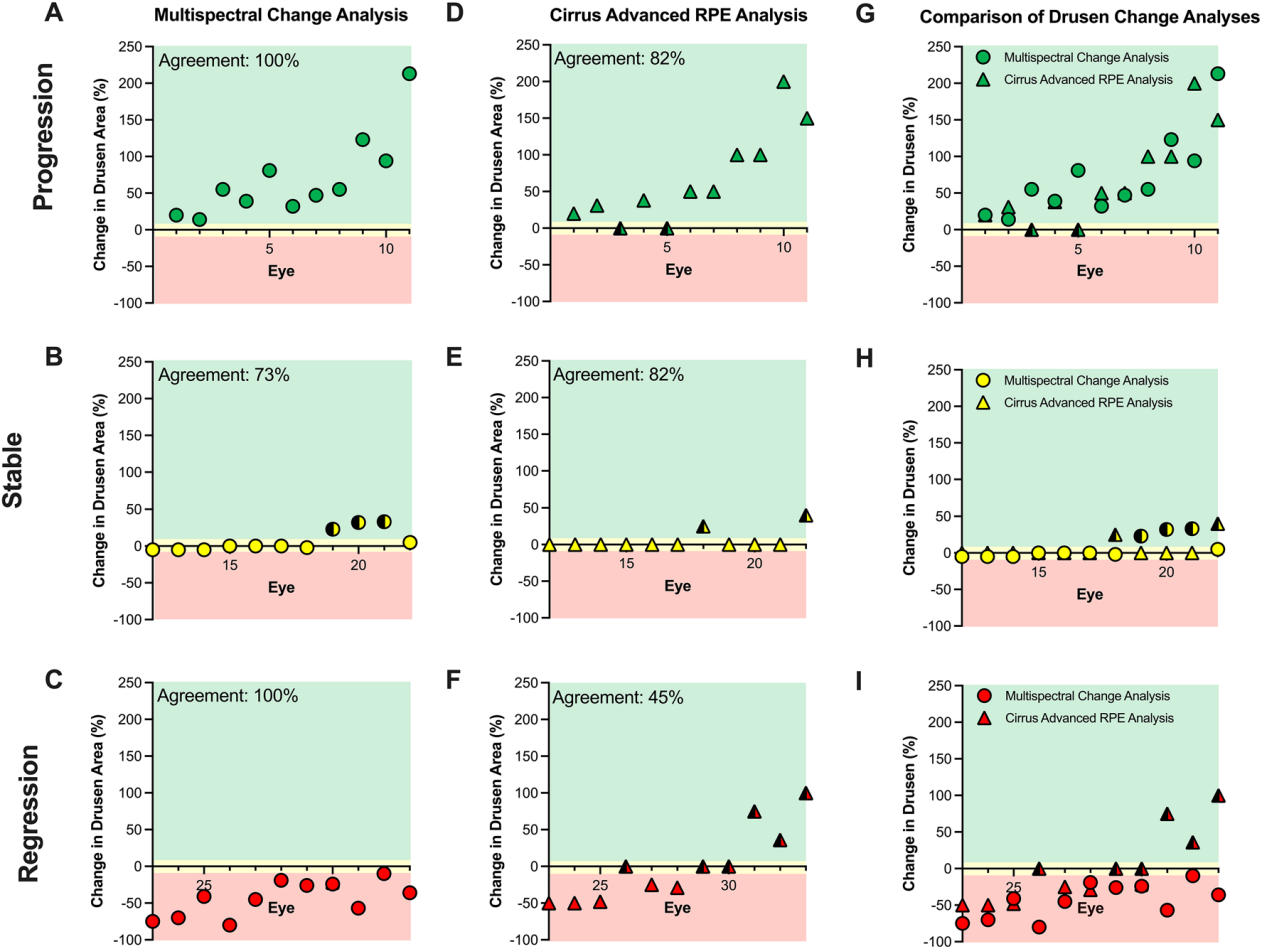
The change in drusen area as assessed using (**A**-**C**) Multispectral pattern recognition change analysis or (**D**–**F**) Cirrus Advanced RPE Analysis. For direct comparison of measurements on the same eye, data from both analyses are also displayed together (**G**–**I**). Data is grouped based on drusen change classification by expert graders: progression (**A**, **D**, **G**), stable (**B**, **E**, **H**) and regression (**C**, **F**, **I**). The background of the plots are shaded green, yellow, and red to correspond to areas classified as progression (> 5% change in drusen area), stable (-5% to 5%) and regression (> -5% change) respectively. False positives/negatives are marked by data points half colored in black.

When all eyes were evaluated against expert graders, multispectral pattern recognition change analysis was in 91% agreement (30/33 eyes) in direction of drusen change and exhibited a sensitivity of 100% (22/22 eyes) with a specificity of 73% (8/11 eyes). When sub-groups were analysed individually, there was 100% agreement between multispectral pattern recognition change analysis and expert graders for eyes classified as having drusen progression (Fig. 4A) and drusen regression (Fig. 4C). Disagreement arose from three eyes which were false positive for drusen progression, demonstrating a 23–33% increase in drusen area according to multispectral pattern recognition change analysis compared to expert graders who indicate no change in drusen area (Fig. 4B).

### Sensitivity and specificity of cirrus advanced RPE analysis to expert graders

Relative to expert graders, the agreement of the Cirrus Advanced RPE Analysis for all eyes was only 70% (23/33 eyes) in direction of drusen change (Fig. 4D–F). Cirrus Advanced RPE Analysis demonstrated lower sensitivity of 64% (14/22 eyes) than Multispectral pattern recognition change analysis but higher specificity of 82% (9/11 eyes).

In sub-group analysis, there was 82% agreement between Cirrus Advanced RPE Analysis and expert graders with the former indicating drusen stability for two eyes when graders suggested there was drusen progression (Fig. 4D). There was 82% agreement between Cirrus Advanced RPE Analysis and expert graders for eyes with stable drusen involvement with two eyes falsely showing drusen progression (Fig. 4E). For eyes with drusen regression, there was only 45% agreement between Cirrus Advanced RPE Analysis and expert graders. Three eyes falsely showed progression and more importantly, three eyes falsely showed stability when graders flagged regression (Fig. 4F).

### Assessing the role of differences in drusen area on analyses outcomes

**Figure 5:**
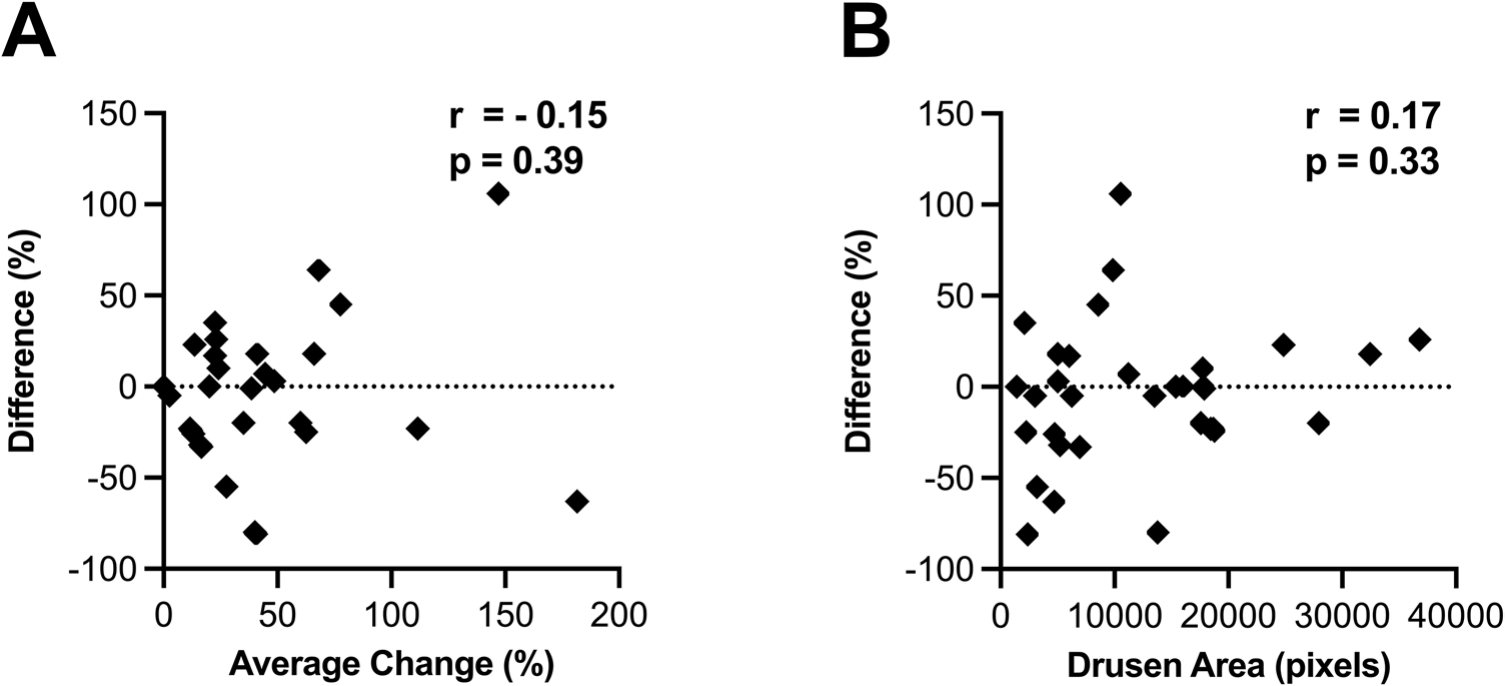
(**A**): Bland–Altman plot of the average change in drusen area and difference in percentage change measured by Multispectral Change Analysis and Cirrus Advanced RPE Analysis shows there is agreement between the drusen change analyses, r = -0.15. (**B**) Correlation value of r = 0.17 suggests there is no relationship between difference in drusen change measured by the analyses and the initial drusen size measured for each eye analysed.

Finally, we examined if the differences between agreement with expert graders and each analysis possibly resulted from differences in drusen area between eyes. Firstly, we assessed if there was a systematic difference in quantification of drusen area by each analysis (i.e. if one of the analysis consistently quantifies greater or less difference than the other). The average difference between percentage change in drusen area for each eye between the two analyses were minimal across all subgroups: −3% ± 41% (−81–106%), −4% ± 8% (−33–35%) and −6% ± 35% (−80–64%) for drusen progression, stable drusen and drusen regression, respectively. Furthermore, there was no significant difference in mean difference between the change analyses between subgroups (*p* = 0.48, one-way ANOVA). A Bland–Altman plot also confirmed agreement between the two analyses (Fig. 5A). This suggests that the difference between each analysis is not likely to be a consistently an over- or under-estimation in change in drusen area by one of the analyses.

Secondly, as there was a large range in total drusen area between eyes, there was a possibility that the difference between the two change analyses were exaggerated in eyes with a large area of drusen (and therefore greater possibility for absolute differences in change) compared to those with only a small area of drusen. To assess this, total drusen area of each eye at the initial visit was assessed relative to the difference in change between each analysis (Fig. 5B). No significant correlation was observed between these two variables (r = 0.17; *p* = 0.33, Pearson’s correlation). Subgroup analysis found no significant correlations (data not shown). Multivariate analysis also determined that no demographic or magnification factors (specifically age [*p* = 0.91] and refractive error [*p* = 0.39]) were confounders in the analyses.

## Discussion

Multispectral pattern recognition change analysis identified drusen progression and drusen regression with a sensitivity of 100% and specificity of 73% compared to expert grading. This was a greater level of agreement than the Cirrus Advanced RPE Analysis to expert grading. Importantly, false positives with multispectral pattern recognition change analysis only occurred in ‘low-risk’ eyes with a stable drusen pattern compared to Cirrus Advanced RPE Analysis where false negatives include mis-classification of eyes with drusen progression and regression as stable (Fig. 4G–I). These results suggest multispectral pattern recognition change analysis is a useful tool for measuring change in drusen area which is correlated to a higher risk of progression to late AMD^4–6^.

### Multispectral pattern recognition outperforms current commercial tools for drusen change analysis

Multispectral pattern recognition provided greater agreement with expert graders for direction of drusen change relative to Cirrus Advanced RPE Analysis. Comparison of the two methods suggested this was not associated with inherent differences in eyes such as the amount of drusen present or the amount of change in drusen area between visits. Considering assessment of drusen change in the literature have been almost exclusively reported using Cirrus software^7,14–18,45^, this result suggests that improvement in automated assessment of longitudinal drusen change could be achieved using a multispectral pattern recognition approach.

Possible reasons for the poorer performance of the Cirrus Advanced RPE analysis may be related to limitations associated with the detection of small drusen as acknowledged by Zeiss^20^. Abdelfattah et al.^21^ highlighted this with 31.5% of participants (28/89 eyes) in their study unable to be assessed as their drusen volume fell below the threshold of measurement by the Cirrus OCT software. Jain et al.^22^ also suggested greater detection of large drusen using OCT and greater detection of smaller drusen using color fundus photography. Inability to detect small drusen may explain why 18% of patients with drusen progression in this study were misclassified as stable by the Cirrus Advanced RPE analysis. More recently, a novel automated RPE elevation algorithm utilising optical coherence tomography angiography images instead of OCT images to provide a more anatomically correct basement membrane “floor” for the drusen measurements has been proposed to improve detection of small drusen^19^.

Other reasons for disagreement of the Cirrus Advanced RPE analysis and expert graders is misdiagnosis of RPE elevations with a post-hoc analysis of 115 subjects from a diversified normative database demonstrating the Advanced RPE analysis identified RPE elevations in up to 6.1% of the normal subjects^14,16,20^. Our previous work indicates that multispectral pattern recognition exhibits low spectral diversity for images of normal eyes, providing strong evidence that the analysis is accurate in identifying AMD-specific lesions such as drusen in AMD eyes only^33^.

## Multispectral pattern recognition can assist in applying a multimodal approach to AMD assessment

Work by Guymer and Wu^12^, has highlighted a multimodal approach to AMD assessment can provide the best clinical management of the disease based on structural markers. However, a multimodal approach potentially complicates assessment in clinical practice by introducing an increased time and labour burden associated with analysing multiple images. The information barriers are likely enhanced when patients are monitored over numerous visits as in typical in AMD^46,47^. Indeed, Ly et al^37^. showed diagnostic accuracy for AMD only improved by 5% in a series of case vignettes assessed by primary eye-care professionals when additional information from multimodal imaging was provided.

Our previous proof-of-concept work using multispectral pattern recognition for multimodal *en face* imaging of AMD demonstrated this technique could detect AMD lesions such as large drusen and pigmentary changes with 74% sensitivity and 98% specificity and non-AMD anatomical features such are blood vessels or normal retinal tissue with 99% and 96% sensitivity, respectively, and 98% specificity^33^. In this study, we further demonstrated that multispectral pattern recognition can detect longitudinal change in drusen area accurately (91% agreement with expert graders). In theory, the accuracy of this method and the presentation of clinical data, single pseudocolor map with a finite set of colors versus multiple images, should assist clinicians in applying a multimodal approach in clinical practice. It may also make interpretation by non-experts more accessible which may open more options for models of care in AMD assessment. Finally, multispectral pattern recognition could also have implications for clinical trials by providing a quantifiable method to assess evolution of drusen over time, a common clinical trial classification criteria and end point^10,11,48,49^. Further work however is needed to confirm AMD lesions observed with this multispectral approach are in agreement with traditional single image modalities (both standalone and in conjunction with each other).

### Multispectral pattern recognition indicates drusen exhibit varying spectral signatures

In this study we found up to three different spectral theme classes associated with drusen in some eyes. This was similar to our previous proof-of-concept study^33^. In many cases, different spectral classes were found to surround each other within the same overall drusen area seen on color fundus photography or OCT. This suggests the different spectral classes likely reflect spectral differences associated with the border and center of drusen which may simply occur from differences in anatomical distortion of the retinal layers at these locations. As such, in this study it was appropriate to pool these spectral theme classes together for quantification.

Different drusen subtypes, notably reticular pseudodrusen, however can also distort outer retinal layers differently to conventional drusen and therefore, it is possible that different spectral theme classes could reflect different drusen subtypes. In this study, expert graders indicated that all eyes only showed signs of conventional drusen and therefore the pooling of multiple spectral theme classes is unlikely to reflect pooling of different drusen subtypes. However, considering subtypes such as reticular pseudodrusen are associated with greater risk of AMD progression than conventional drusen^50,51^, identifying and following changes in these lesions through specific spectral theme classes could have high prognostic value. We have yet to explore the role of multispectral pattern recognition in eyes with confirmed different drusen subtypes to see if this is the case.

### Limitations

This study was limited by a sample size of 33 eyes with a follow up time of approximately 1.3 years. However, we noted consistent findings and low variability amongst the study cohort with regards to the difference observed between analyses even though our sample covered a wide range of drusen area sizes and directions of drusen change (i.e. progression and regression). The time frame between visits also holds clinical relevance in assessing drusen change analyses as it is the recommended time for follow-up for individuals with intermediate AMD^43,44^. These results suggest the outcomes from this study are likely an adequate reflection on the performance of multispectral pattern recognition for change analysis in intermediate AMD.

In addition, our gold standard measurement was the clinical diagnosis of two expert graders. These graders had access to multimodal imaging of eyes and therefore could use a multimodal approach to their diagnosis. As such, these diagnoses could have biased towards supporting a similar methodology (i.e. multispectral pattern recognition) versus a single modality approach (Cirrus Advanced RPE Analysis). This method was chosen as it corresponds to the current standard of care^12^ and therefore ensures the results of this work are in context with current clinical practice^33^.

Finally, multispectral pattern recognition provides area of drusen but not volume. This suggests that if changes in drusen over time exclusively affected drusen height and not area, they may not be detected by our method. Interestingly however we demonstrated that multispectral pattern recognition is able to generate different spectral theme classes for the center and edge of drusen suggesting drusen height possibly be identified through this method. Future studies are currently underway exploring different spectral theme classes of drusen in relation to drusen cross-sectional characteristics on OCT which may allow for better definition of spectral theme classes and expansion of change analysis.

## Conclusion

This study demonstrates that multispectral pattern recognition of various *en face* retinal imaging modalities can accurately quantify longitudinal change in drusen area in eyes with intermediate AMD. This analysis was in greater agreement with expert grading than a commercially available change analysis tool, the Cirrus Advanced RPE analysis regardless of the direction of drusen change. Considering the association of drusen change and AMD progression, multispectral pattern recognition could be a highly useful tool in assessing drusen in clinical practice and other contexts such as clinical trials.
